# Effects of Smoking Cannabis on Visual Function and Driving Performance. A Driving-Simulator Based Study

**DOI:** 10.3390/ijerph17239033

**Published:** 2020-12-03

**Authors:** Sonia Ortiz-Peregrina, Carolina Ortiz, José J. Castro-Torres, José R. Jiménez, Rosario G. Anera

**Affiliations:** Laboratory of Vision Sciences and Applications, Department of Optics, University of Granada, 18071 Granada, Spain; soniaortiz@ugr.es (S.O.-P.); jjcastro@ugr.es (J.J.C.-T.); jrjimene@ugr.es (J.R.J.); rganera@ugr.es (R.G.A.)

**Keywords:** cannabis, vision, simulator, impaired driving, driving safety

## Abstract

Cannabis is the most widely used illegal drug in the world. Limited information about the effects of cannabis on visual function is available, and more detail about the possible impact of visual effects on car driving is required. This study investigated the effects of smoking cannabis on vision and driving performance, and whether these effects are correlated. Twenty drivers and occasional users were included (mean (SE) age, 23.3 (1.0) years; five women). Vision and simulated driving performance were evaluated in a baseline session and after smoking cannabis. Under the influence of cannabis, certain visual functions such as visual acuity (*p* < 0.001), contrast sensitivity (*p* = 0.004) and stereoacuity (far, *p* < 0.001; near, *p* = 0.013) worsened. In addition, there was an overall deterioration of driving performance, with the task of keeping the vehicle in the lane proving more difficult (*p* < 0.05). A correlation analysis showed significant associations between driving performance and visual function. Thus, the strongest correlations were found between the distance driven onto the shoulder and stereoacuity, for near (*ρ* = 0.504; *p* = 0.001) and far distances (*ρ* = 0.408; *p* = 0.011). This study provides the first evidence to show that the visual effects of cannabis could impact driving performance, compromising driving safety. The results indicate that information and awareness campaigns are essential for reducing the incidence of driving under the influence of cannabis.

## 1. Introduction

Today, driving under the influence of cannabis (DUIC) is one of the major concerns in terms of road safety. THC (Δ9–tetrahydrocannabinol), the main psychoactive compound of cannabis, is the most-often detected illicit substance in drivers [1]. In Spain, roadside studies have revealed that more than half of drivers who tested positive for drugs had consumed cannabis [2,3]. The same trend has been found in countries such as the United States and Canada, where the prevalence of DUIC has also increased in the past years [4,5].

There is evidence that cannabis use has a negative effect on driving, increasing the accident risk [6]. The systematic review and meta-analysis conducted by Asbridge et al., (2012) concluded that cannabis consumption almost doubles the collision risk, particularly for fatal collisions [7]. With regard to driving performance, cannabis use has been shown to have a negative influence on certain aspects, like driving stability, as drivers under the influence of cannabis maintain their lane position less well [8,9]. Likewise, longitudinal control seems to be affected by cannabis use, with drivers under the effects of cannabis driving more slowly and adopting longer following distances relative to a lead vehicle [10].

Undoubtedly, cannabis impairs some functions needed for driving, such as cognitive or motor skills. Sensory systems are also essential for driving, with vision being the main sensory mechanism involved in this task. There is, however, little evidence about the effects of cannabis consumption on vision. A limited number of studies have addressed this issue, demonstrating that cannabis users seem to suffer significant impairments in aspects such as color vision [11] and visual information processing [12,13,14]. A more complete visual status evaluation was performed by Dawson et al., (1977), who reported that, after ten years of cannabis use, participants showed increased intraocular pressure, longer dark-adaptation periods, reduced visual acuity, reduced color discrimination, and increased lacrimation [15]. Likewise, significant impairments in dynamic visual acuity have been reported [16], as well as for ocular movements in reading tasks [17].

More recently, studies have found that cannabis significantly impairs other visual parameters such as contrast sensitivity [18,19], which has been identified as an important predictor of safe driving [20]. As stated above, a great body of evidence demonstrates that a significant number of drivers use their car when under the influence of this substance [3,4,5], so the visual changes induced by its use could have serious safety implications.

Taken together, these findings suggest that cannabis use could impact visual functions, and that other non-standard visual parameters should be investigated. In addition, it is important to understand the magnitude of the visual effects caused by acute cannabis intoxication, and how these influence tasks that are highly dependent on visual information, such as driving. The aim of this work was, therefore, to evaluate the effects of cannabis use on vision for a wide range of visual parameters and on driving performance. We also investigated whether the effects of cannabis smoking could be correlated with worse driving performance as a first step towards studying the implications of cannabis consumption for safety risk while driving.

## 2. Materials and Methods 

### 2.1. Participants

Twenty volunteers were included in the study (mean age (SE), 23.3 (1.0) years; range 19–36 years), of which five were women. All the subjects were occasional cannabis users, i.e., self-reported cannabis consumption of at least once but less than four times/week over the preceding three months [9,10]. Other inclusion criteria were: a current driver’s license with at least one year of driving experience; driving at least once a week; monocular visual acuity of at least 6/6 (Snellen notation) using any habitual correction for driving and normal binocular vision. The exclusion criteria included certain past or current medical illness: current cannabis or alcohol use disorders identified by the Cannabis Use Disorders Identification Test-Revised (CUDIT-R) [21] and/or the Alcohol Use Disorders Identification Test (AUDIT) [22]; the use of other drugs; pregnancy or breastfeeding; and simulator sickness.

This study was in line with the Declaration of Helsinki and was prospectively approved by the University of Granada Human Research Ethics Committee (921/CCEIH/2019). Prior to participating, the subjects were verbally informed about the details and possible consequences of the study, and a signed informed consent was obtained from each participant.

### 2.2. Visual Assessment

High-contrast static visual acuity was assessed binocularly at 5.5 m using the chart implemented in the POLA VistaVision Visual Chart System (DMD Med Tech srl. Torino, Italy). Binocular contrast sensitivity was also measured, using the same device, in order to analyze the sensitivity of the visual system not only for size but also for contrast. For this purpose, we measured the contrast threshold (i.e., the minimum contrast required to see a visual grating target over a uniform background). The inverse of the contrast threshold is the contrast sensitivity for a given frequency. The spatial frequencies tested were: 0.75, 1.5, 3, 6, 12 and 18 cpd (cycles per degree), each one with eight contrast levels, at a distance of 2.5 m.

Stereoacuity (i.e., the capacity to distinguish the spatial or three-dimensional location of objects in the environment) was also studied as a key visual function for driving. For this purpose, we evaluated two testing distances. For far-distance testing, we employed the stereotest implemented in the VistaVision monitor at 5.5 m. The stereotest can measure disparities ranging from 300 to 10 arcsec using rows of five polarized vertical lines at each level of disparity assessed. For near-vision testing, we employed the Fly Stereo Acuity Test with LEA Symbols (Stereo Optical Co., Inc., Chicago, IL, USA), a polarized-based test that contains a graded circle test, evaluating disparities from 400 to 20 arcsec.

Pupil size was also recorded using the NeurOptics^®^ VIP^®^-300 pupillometer (NeurOptics, Irvine, CA, USA), based on infrared technology. Pupil size was measured at different background illuminations simulating scotopic (background is off), low mesopic (0.3 lux) and high mesopic (3 lux) viewing conditions. Low mesopic simulates lighting conditions such as moon illumination, driving at night outside urban areas, or a dimly lit room; while high mesopic simulates conditions such as moderate streetlighting or early twilight.

The accommodative response (AR) is defined as the ocular ability to see clearly at different fixation distances and the accuracy required to maintain a steady level of focus at the distance [23]. If the AR is lower than the accommodative demand, this is called accommodative lag, while in the opposite case, where the AR is higher than the accommodative demand, is called accommodative lead. The AR was studied by means of an open-field autorefractor, the Grand Seiko WAM-5500 (Grand Seiko Co. Ltd., Hiroshima, Japan), a clinically validated refraction tool [24]. For testing, we employed the static mode and the fixation target used was a 2 cm high-contrast black star (Michelson = 79%) printed on a white background. The subjects had to look at the target binocularly through a 12.5×22 cm open-field beam-splitter, although the autorefractor is only able to record data from one eye at a time. Firstly, an initial measurement was taken at a viewing distance of 6 m (baseline refraction value), the measurement being repeated nine times. Then, the target was placed at 0.4 m and 0.2 m, the two accommodative demands studied, and nine measurements were taken for each distance. During data acquisition, the participants had to rest their forehead and chin on the head/chin support and the subject’s alignment with the fixation target was checked to ensure on-axis measurements. The accuracy of the AR was obtained as in Poltavski et al., (2012), by subtracting the mean spherical equivalent of the nine measures and the baseline refraction value (spherical equivalent) from the accommodative demand required by the target distance (2.5 or 5D) [25].

All the tests performed in the visual assessment were carried out while the participants were wearing the spectacle correction usually worn for driving (if any).

### 2.3. Driving Assessment

The software employed for the simulation was SIMAX DRIVING SIMULATOR v.4.0.8 BETA (SimaxVirt S.L., Pamplona, Spain). This is a fixed-base driving simulator with a 180° field of view. The participants drove an itinerary of approximately 12.5 km, which required about 15 min to complete when abiding by the speed limits established. The driving scenario was performed in daylight and under good weather conditions, and consisted of three main sections. The first was 4.5 km of dual carriageway (with two lanes of traffic in each direction), with a 120 km/h speed limit, no buildings, moderate traffic, and a gentle curve. The second section was 6 km of one-lane single carriageway winding mountain road, with a variable speed limit of 40–90 km/h, no buildings, and moderate traffic. The third section was a 2 km-long inner-city circuit, a variable speed limit of 40–50 km/h, several intersections or roundabouts with traffic signals, many buildings, pedestrians, and moderate traffic. More information on the driving simulator can be found in other works [26,27,28,29,30]. 

To assess simulated driving performance, we considered the variables indicated in Table 1. The Overall Driving Performance Score (ODPS) was determined as in other works [28,30,31], by obtaining z-scores for individual driving parameters and calculating a mean z-score for each participant. Positive ODPS represent a better performance than the mean.

Reaction time was based on braking response. To measure this parameter, the simulator generated three braking events in different random positions along the mountain road. On this part of the route, drivers commonly have to follow other vehicles that are in front of them. Under these circumstances, when the driver reaches a sufficient speed, the simulator generates a braking event in which the car in front of them makes a sharp braking stop. For these events, the reaction time was calculated as the difference between the instant at which the car in front turned on its brake light, and the instant at which the test subject pressed the brake pedal.

### 2.4. Procedure

Participants underwent two training sessions that lasted about 15 min and involved a similar scenario to those used in the experimental drives. At the end of the training sessions, all the subjects said they felt totally comfortable with the equipment. Then, the two experimental sessions (one involving no substance use, and another 20 min after smoking a cannabis cigarette) were conducted in a random order and at least one week apart. Participants prepared the cannabis cigarette as they usually do for their habitual consumption, and they smoked it in about 10 min. In each session, the participants carried out the visual tests and followed the route in the driving simulator, also in a random order. The testing sessions lasted about 75 min, guaranteeing a considerable psychoactive effect during the session after smoking cannabis, given that this tapers off within 2–3 h [32]. All the participants were asked to abstain from alcohol for the 24 h prior to each testing session and from cannabis for four days. To obtain an objective drug-intake screening during the sessions, a saliva drug test was performed using the Dräger DrugTest 5000 (Dräger Safety AG & Co. KGaA. Lübeck, Germany). This device has been proposed as a highly sensitive, specific and efficient method for detecting cannabinoids in oral fluid [33]. The test is able to verify the consumption of cannabis, amphetamines, benzodiazepines, cocaine, methamphetamines, opiates, methadone, and ketamine. In the case of cannabis, the Draeger test is able to detect concentrations higher than 12 ng/mL up to 8–14 h after consumption. To check that the participants had not consumed alcohol, we also measured their breath alcohol content (BrAC) with the Dräger Alcotest 7110 MK–III (Dräger Safety AG & Co. KGaA. Lübeck, Germany).

### 2.5. Statistical Analysis

Descriptive statistics, including the means and standard errors (SEs) for sociodemographic, vision and driving variables were computed. To compare visual parameters and driving performance variables between testing conditions (baseline and after smoking THC), the paired t-test and Wilcoxon test were applied, when necessary, according to the data distribution (Kolmogorov–Smirnov test). Reaction time is associated with other factors related to driving that may be affected by smoking cannabis, including decision making, cognition, and concentration. For this reason, we studied the correlations between reaction time and the other driving performance parameters (Spearman correlations). Likewise, a Spearman correlation analysis was run to study the associations between visual function parameters and driving performance variables.

## 3. Results

**Figure 1:**
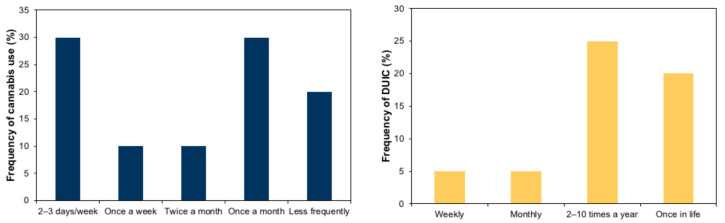
Self-reported frequency of cannabis use during the last six months (left) and self-reported frequency of DUIC (right) among the participants of the study.

Participants indicated a mean (SE) driving frequency of 4.5 (0.5) days per week, and a mean (SE) driving experience of 4.1 (0.9) years (range 1–17 years). The mean (SE) age when they first used cannabis was 17.6 (0.4) years. The mean (SE) score for the AUDIT questionnaire was 7.1 (0.8) (max. score 40 points) and 6.1 (0.9) for the CUDIT-R (max. score 32 points). Figure 1 shows the results of self-reported data about frequency of cannabis use and frequency of DUIC. The total percentage of self-reported DUIC was 55% and, worryingly, 15% reported they had even smoked cannabis while driving.

### 3.1. Visual Assessment

**Figure 2:**
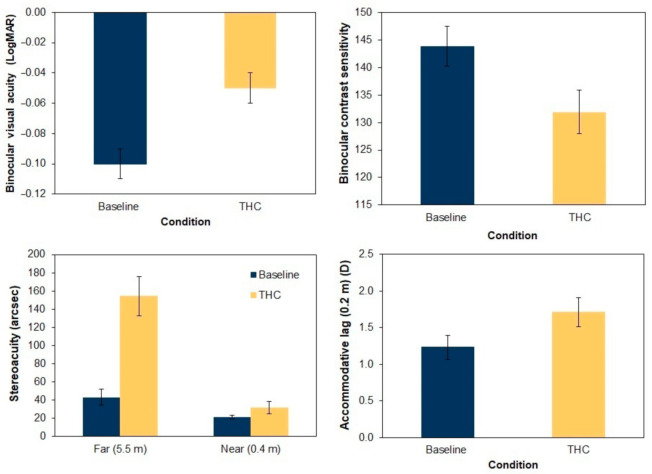
Results obtained for visual parameters that deteriorated significantly after smoking cannabis (*p* < 0.05).

Our analysis indicated significant differences in visual acuity, with a reduction of approximately two lines in the cannabis condition compared to the baseline condition (*p* < 0.001) (see Table 2 and Figure 2). Stereoacuity was also significantly deteriorated, for both the distances evaluated, although the most notable worsening was for distance vision, with an average increase of 111 arcsecs (*p* < 0.001).

Moreover, pupil size for the scotopic condition decreased an average of 0.32 mm (*p* = 0.008) after smoking cannabis, although in mesopic conditions the pupil size remained very similar. The average contrast sensitivity was reduced 8.3% after taking THC, this deterioration being significant for the spatial frequencies of 0.75 cpd, (*p* = 0.04) and 12 cpd, (*p* = 0.02). Finally, the accommodation accuracy was reduced in comparison to the baseline condition. The participants exhibited greater accommodative lags after smoking cannabis (29% and 39% more at 0.4 and 0.2 m, respectively), although the differences were only significant at 0.2 m (*p* = 0.01).

### 3.2. Driving Assessment

Cannabis consumption also caused impairment in driving performance, as shown in Table 3. On the dual carriageway, the participants drove 2.5% faster under the effects of cannabis and also travelled 71.9% further onto the shoulder, although neither of the two variables was significantly different with respect to the baseline condition.

On the mountain road, just like on the dual carriageway, no significant increase in speed was found after smoking cannabis. However, the participants did show greater difficulties staying in their lane, and they travelled a greater distance invading the opposite lane and the shoulder, resulting in a 50.7% increase in the total distance travelled outside the lane (Table 3). Likewise, the SDLP also increased significantly after cannabis use, indicating that, after smoking, the drivers showed a greater tendency to weave within their lane. The braking reaction time was 0.08 s greater after smoking cannabis than for baseline condition, but this difference was not significant.

In the city, participants driving under the influence of cannabis exhibited a slightly higher mean speed, but the differences did not reach statistical significance. Finally, the participants took less time to complete the entire circuit after smoking cannabis and the number of collisions was higher than in the baseline condition. Overall, cannabis use negatively affected driving performance, as demonstrated by the reduction in the overall driving performance score (ODPS).

In addition to vision, cannabis smoking may impair other factors related to driving, including decision making, cognition and concentration. One of the variables strongly related with such aspects is reaction time. However, the results obtained for reaction time showed no significant correlation with the other driving performance variables, except for the ODPS (*ρ* = 0.442; *p* = 0.005), given that reaction time was employed to calculate this variable.

### 3.3. Relationship between Visual Parameters and Driving Performance

Correlation analysis revealed that the strongest associations were between the distance driven onto the shoulder on the mountain road and stereoacuity for near (*ρ* = 0.504; *p* = 0.001) and far distances (*ρ* = 0.408; *p* = 0.01). The near-distance stereoacuity also proved to correlate significantly with a greater distance travelled invading the shoulder on the dual carriageway (*ρ* = 0.373; *p* = 0.02) and with a greater SDLP (*ρ* = 0.315; *p* = 0.048), indicating that good stereoacuity assists in controlling the vehicle within the lane, allowing the distances between the car and the road boundaries to be judged more accurately. Other significantly associated parameters were visual acuity with the SDLP (*ρ* = 0.372; *p* = 0.02), and contrast sensitivity with the distance travelled invading the shoulder on the mountain road (*ρ* = −0.377; *p* = 0.02). Finally, it should be noted that the ODPS was significantly associated with visual acuity, hence worse visual acuity meant worse driving performance (*ρ* = −0.370; *p* = 0.02).

## 4. Discussion

The data presented suggests that smoking THC significantly impairs the driving performance of users, principally their ability to stay in their driving lane. In previous work, the SDLP has also been shown to be a validated measure of the degree of driving impairment associated with THC use [8,9,34]. In agreement with the scientific literature, deteriorated driving stability increased the distance travelled outside the lane [35], an aspect of particular relevance on narrow or winding roads with oncoming traffic, such as interurban or rural roads, as this could increase the risk of accidents. 

Despite this, in our study collisions did not increase significantly, confirming the finding of an earlier simulator-based study [35]. Two meta-analyses of observational epidemiological studies have found that cannabis use doubles the risk of crashes [7,36]. However a subsequent meta-analysis including, among others, the two studies mentioned above, did not find this association [37], so this question requires further research.

Our results support previous studies, since we found that smoking THC does not induce a significant increase in reaction time [34]. The overall rate of driving performance deteriorated significantly with cannabis use, contrary to a study that employed another type of general score, based in driving error variables, to compare driving performance under the effects of cannabis, alcohol and both substances [35]. This difference with our ODPS result could be due to the type of variables included, so it would be advisable to consider the aspects of driving that are most sensitive to cannabis use, such as SDLP.

The majority of papers focusing on the visual consequences of cannabis use claim that these could pose a danger to driving [18,19], although, to the best of our knowledge, no studies have correlated these two aspects. Vision is essential for driving, and the findings of this study suggest that this sensory mechanism is affected by cannabis use. The use of this substance significantly reduced static visual acuity, contrary to the findings of Adams et al. (1975), however, those authors did find reductions in dynamic visual acuity with alcohol and cannabis [16,38]. Other authors have suggested that cannabis could generate blurred vision [39]. Our results support this idea and, in addition, visual acuity has been significantly correlated with general driving performance (ODPS). The driving environment comprises different stimuli and its dynamics makes them change constantly, presenting different sizes and contrasts. Previous studies on subjects with altered visual acuity have found a relationship between deterioration of this visual parameter and worse performance in signal and obstacle detection during driving [40], and SDLP [41]. We also found a significant correlation between poorer VA and increased SDLP. Deteriorated visual acuity after cannabis use could affect the correct integration of visual information from the road, thus worsening driving stability. Visual acuity allows a driver to correctly discriminate between stimuli on the road; however, the spatial location of stimuli is also critical to driving. This issue is confirmed by our findings with regard to stereoacuity, which, in addition to significantly worsening after cannabis smoking, has been shown to significantly correlate with several driving-related parameters (SDLP and distance driven outside the limits of the lane). Although there is no previous data on the effect that THC has on stereoacuity, several studies have reported that this drug influences a phenomenon related to three-dimensional vision, the binocular depth inversion illusion (BDII) [42]. BDII results have shown a reduction in the occurrence of this optical illusion under the effect of cannabis [14], as well as in regular cannabis users [12].

Our results show that contrast sensitivity is significantly correlated with the distance driven invading the shoulder. Under the effects of cannabis, our participants showed an 8% mean reduction in CS. This result is in line with recent studies that have shown permanent CS impairment in cannabis users [18,19]. The evidence therefore suggests that cannabis consumption impacts low level visual processing, perhaps due to the presence of CB1 cannabinoid receptors on the retina [43]. Previous work supports the idea that CS is very important for driving. Thus, in drivers with diminished CS, problems in recognizing road stimuli are emphasized [31], and our recent work has also found a significant correlation between poorer contrast sensitivity and worse SDLP [26,28].

Although the accuracy of the accommodative response decreased after smoking cannabis, we did not find a correlation with driving performance parameters. This could be due to the fact that smoking cannabis significantly altered accommodation only at the nearest distance (0.2 m), a less-relevant distance for driving. Finally, we found that smoking THC significantly reduced pupil size in the scotopic level. Brown et al., (1977) also reported smaller pupil sizes at low-photopic light levels [44], but other studies have shown increased sizes as well [45]. This question must therefore be clarified in order to discuss its potential effect on driving performance, especially for nighttime driving.

This study is not without limitations, and we must consider these when interpreting the results. Firstly, the use of a driving simulator supposes an important limitation due to its inability to completely represent the realism of the driving environment. Even though the fidelity of driving simulators is becoming more and more realistic, these do not have the variety that we can find in the real world. For instance, the simulator did not included road stimuli with reduced contrast, which can be very common in real driving conditions. Future studies should include road stimuli that comprise different complexity due to its visibility in order to determine whether cannabis’ visual effect could represent more risk in these circumstances. Nevertheless, it is important to note that this simulator has been used successfully in previous studies [26,28,30] and there is evidence to support the relative validity of driving simulators with respect to actual driving [46].

Secondly, the participants included in the sample were only occasional users. In order to determine if regularity of cannabis use determines differences in the effect on visual function, future studies should include more regular users.

Thirdly, the fact that each participant consumed the cannabis cigarette as they usually do, does not allow us to establish a relationship between dose or blood concentration and effect. However, the relationship between dose, blood concentration, and effect is non-linear [32,47]. Our goal was to study the consequences that a participant’s typical use, on a normal day, has on vision and driving, and thus be able to relate these two aspects from a real perspective. In fact, an important proportion of the participants indicated that they had driven under these circumstances. These results should serve as a starting point for future work.

Finally, it is important to note that correlation does not imply causation. Although we have found statistical associations between visual parameters and driving performance variables, there are other factors independent of vision that could account for the deterioration in driving ability, such as impairment of cognitive or motor functions. The results we have presented indicate that visual impairment could be of interest when considering driving under the influence of cannabis, but future work that also takes into account other influential factors is needed to confirm our findings.

## 5. Conclusions

Cannabis consumption has a negative effect on both visual function and driving performance. In part, the impairment noted in driving performance could be due to the visual degradation, given that most of the integrated information for driving is captured by the visual system. We have found significant correlations between certain visual and driving-performance parameters, particularly regarding driving stability. Thus, our results highlight the importance of parameters such as visual acuity, contrast sensitivity or stereoacuity, which play a key role in maintaining the vehicle in the lane properly. Moreover, our results suggest a lack of awareness of the risks associated with cannabis use in driving, given that a considerable proportion of participants have driven after smoking cannabis. There is, therefore, still a considerable need for awareness-raising and information campaigns aimed at the general public, as well as for research that provides adequate insights into how this drug affects both short- and long-term vision and the ability to drive safely.

## Figures and Tables

**Table 1:** Variables considered for assessing driving performance in the study.

Section of the Route	Driving Performance Variables
Dual carriageway	Mean speed (kph) Distance travelled onto the shoulder (m) ^1^
Mountain road	Mean speed (kph) Distance driven invading the opposite lane (m) Distance driven onto the shoulder (m) Total distance driven outside the lane (i.e., the distance travelled onto the shoulder plus the distance travelled invading the opposite lane) (m) ^1^ Standard deviation of the lateral position (SDLP) (m) ^1^ Reaction time (s) ^1^
City	Mean speed (kph)
Total circuit	Total time (s) Collisions Overall Driving Performance Score (OPDS)

^1^ Individual variable selected to compute the OPDS.

**Table 2:** Comparison of visual parameters in the baseline session and after smoking cannabis.

Visual Parameter	Baseline Condition	THC Condition	*t*/Z	*p* Value
Binocular VA (logMAR)	−0.10 (0.01)	−0.05 (0.01)	−4.516	<0.001
Binocular CS	143.85 (3.64)	131.93 (4.01)	3.223	0.004
Stereoacuity- far (5.5 m) (arcsec) ^1^	43.16 (8.89)	154.21 (21.73)	−3.623	<0.001
Stereoacuity-near (0.4 m) (arcsec) ^1^	21.18 (2.11)	31.68 (6.99)	−2.496	0.01
Pupil size (scotopic) (mm)	6.65 (0.18)	6.33 (0.15)	2.94	0.008
Pupil size (low mesopic) (mm)	5.91 (0.19)	5.93 (0.16)	−0.156	0.87
Pupil size (high mesopic) (mm)	5.39 (0.18)	5.43 (0.20)	−0.271	0.79
Accommodative lag (0.4 m) (D)	1.07 (0.17)	1.38 (0.13)	1.949	0.07
Accommodative lag (0.2 m) (D)	1.23 (0.16)	1.71 (0.20)	2.892	0.01

Values are presented as mean (SE); ^1^ Wilcoxon rank sum test; abbreviations: VA, visual acuity; CS, contrast sensitivity; *t*, t-test statistic; Z, Wilcoxon rank sum test statistic.

**Table 3:** Comparison of driving performance variables in the baseline session and after smoking cannabis.

Driving Parameter	Baseline Condition	THC Condition	*t*/Z	*p* Value
Dual Carriageway				
Mean speed (km/h)	115.54 (1.59)	118.47 (2.44)	−1.515	0.15
Distance driven onto the shoulder (m) ^1^	71.44 (18.89)	122.83 (23.54)	−1.61	0.11
Mountain Road				
Mean speed (km/h)	55.08 (0.35)	55.86 (0.39)	−1.77	0.09
Distance driven invading the opposite lane (m) ^1^	322.09 (49.62)	458.87 (115.12)	−1.493	0.14
Distance driven onto the shoulder (m) ^1^	16.96 (3.56)	52.14 (15.44)	−3.219	0.001
Total distance driven outside the lane (m) ^1^	339.04 (49.86)	511.01 (112.27)	−1.979	0.048
SDLP (m)	0.52 (0.02)	0.57 (0.02)	−3.10	0.006
Reaction time (s) ^1^	0.84 (0.03)	0.92 (0.04)	−0.312	0.76
City				
Mean speed (km/h)	29.34 (1.05)	29.94 (1.03)	−0.66	0.52
Total circuit				
Total time (s)	794.38 (12.12)	784.61 (10.84)	0.906	0.38
Collisions ^1^	0.50 (0.14)	0.70 (0.26)	−1.068	0.29
Overall Driving Performance Score ^1^	0.21 (0.13)	−0.29 (0.12)	−3.114	0.002

Values are presented as mean (SE); ^1^ Wilcoxon rank sum test; abbreviations: SDLP, standard deviation of the lateral position; *t*, t-test statistic; Z, Wilcoxon rank sum test statistic.
